# “I Am a Son of the Red Earth”

**DOI:** 10.3201/eid2208.AC2208

**Published:** 2016-08

**Authors:** Byron Breedlove, Frank J. Sorvillo

**Affiliations:** Centers for Disease Control and Prevention, Atlanta, Georgia, USA (B. Breedlove);; UCLA School of Public Health, Los Angeles, California, USA (F.J. Sorvillo)

**Keywords:** art science connection, emerging infectious diseases, art and medicine, about the cover, Hill, Cândido Portinari, I Am a Son of the Red Earth, modernism, parasites, parasitic diseases, Chagas disease, public health

**Figure:**
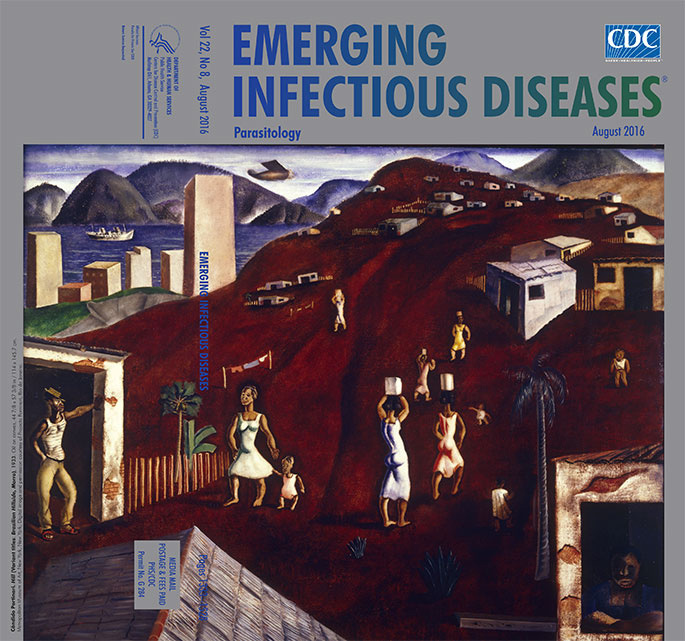
**Cândido Portinari (1903–1962). Hill (Variant titles: Brazilian Hillside, Morro), 1933. Oil on canvas, 44 7/8 × 57 3/8 in/114 × 145.7 cm.** Metropolitan Museum of Art, New York, New York; Digital image and permission courtesy of Projeto Portinari, Rio de Janeiro.

Cândido Portinari, one of Brazil’s most significant artists, was born on the Fazenda Santa Rosa coffee plantation in Brodowski in upstate São Paulo in 1903, the second of 12 children from Italian immigrants from the Veneto region of Italy. Because of his family’s poverty, he did not complete his primary education.

His skill and interest in painting and drawing, evident from an early age, led Portinari to begin his formal training in painting and composition at the National School of Art in Rio de Janeiro in 1919. At the age of 15, he was among the first Brazilian artists to incorporate Modernist elements into his painting, and these elements defined his subsequent works. In 1928, Portinari won a prize at the National Salon of Brazil, which provided funds that enabled him to spend 3 years in Europe, where he traveled extensively, studied European art, visited museums, and met other artists. He continued exploring Modernism and was particularly drawn to Cubism and Surrealism. While in Europe, Portinari also met a young Uruguayan women, Maria Martinelli, his future wife.

After Portinari returned to Brazil in 1931, the artist began, according to journalist Warren Hoge, depicting scenes and themes “covering the country's earliest history, its slave trade, small-town life, gold prospectors, farming, construction, religious processions, circuses, jungle wildlife, urban slums, racial mixture and backlands bandits.” Early in his life, Portinari had witnessed and experienced poverty and inequity, which not only influenced his art but also spurred him to enter politics as well. Tellingly, as noted in a *New York Times* article announcing his death, Portinari once said, “I am a son of the red earth. I decided to paint the Brazilian reality, naked and crude as it is.”

Portinari’s impressive career was marked by his vast output of nearly 5,000 works of art and a lengthy list of prestigious awards and international exhibitions. Although Portinari is considered to be the greatest Brazilian artist, only a small number of his paintings are actually on public display. Portinari died on February 6, 1962, of the toxic effects of the lead-based paints he used when he was preparing an exhibit with about 200 works invited by Milan City Hall (Italy).

This month’s cover image, *Hill*, portrays common people living in a favela in Rio de Janeiro by the sea. Portinari depicts the scene on coffee-colored ground, devoid of most greenery. The worn houses have open doors and windows, a few have small fences. The women trudge up and down the hill, balancing the water they have fetched for the households, their bright pastel dresses belying the severity of their lives. Several young children wander about them. No other children are scampering up the hill, and no other people are seen working. In the lower right of the painting, a woman rests her arms on the window and stares straight at the viewer, conveying through her empty expression the world-weary existence of life in the margins, her visage in contrast with the carefree posture of the man in the tilted hat leaning against a doorway.

It’s likely many such villagers worked in menial occupations in the neighboring city. Portinari’s painting emphasizes the poverty and marginalization by revealing a tantalizing glimpse of the city. Its monolithic buildings jut skyward near blue water surrounded by mountains but physically separated from the dwellings on the hill by a deep trench that gives way to grass that clearly is greener on the other side. Modern buildings, a passenger ship, and an airplane indicate commerce, bustle, and travel.

During the 1930s, health and healthcare would also be concerns among the people of this shantytown. Residents of resource-poor communities anywhere in the world, whether in a peri-urban area or a rural village, often suffer from inadequate housing, lack of education, poor nutrition, impaired immunity, and limited access to healthcare.

Those factors can promote the spread and amplify the global burden of key parasitic diseases, including malaria and lymphatic filariasis, as well as the neglected parasitic diseases, including Chagas disease, cysticercosis, and toxoplasmosis in resource-poor settings. Parasites capable of spreading zoonotic disease, including *Adenocephalus pacificus*, *Baylisascaris procyonis*, *Onchocerca lupi,* and pentostomes, continue to routinely emerge. Other emerging and reemerging infections, including neglected parasitic diseases, exact a staggering human and economic toll and are often linked to poverty and desperate living conditions.

Nearly 9 decades since Portinari painted this shantytown overlooking the city of Rio de Janeiro, economic development, social policies, and public health efforts have vastly reduced deaths from infectious diseases, including those from neglected parasitic diseases, in Brazil, where the average life expectancy is now 75 years. Successes there and in other parts of the world have brought many countries to the point where some parasitic infections—including Guinea worm disease, cysticercosis, and lymphatic filariasis—may be potentially controlled, eliminated, or eradicated through public health interventions. Other parasitic infections may not be eliminated, but their impact on health may be lessened through public health control and prevention efforts. With sufficient resources and resolve, stark human landscapes portrayed in works for art—such as the one illuminated in Portinari’s painting of the *Hill*—and parasitic diseases, may become more rare. 
