# Correction: Vol. 22, No. 8

**DOI:** 10.3201/eid2209.C22209

**Published:** 2016-09

**Keywords:** errata, erratum, correction

The name of Project Portinari, Rio de Janeiro, was misspelled in our August issue. The error has been corrected online (http://wwwnc.cdc.gov/EID/article/22/8/AC-2208_article).

